# The Role of Photoactivated and Non-Photoactivated Verteporfin on Tumor

**DOI:** 10.3389/fphar.2020.557429

**Published:** 2020-10-15

**Authors:** Changran Wei, Xiangqi Li

**Affiliations:** ^1^Department of The First Clinical Medical School, Shandong University of Traditional Chinese Medicine, Jinan, China; ^2^Department of Breast Surgery, The Second Affiliated Hospital of Shandong First Medical University, Tai’an, China

**Keywords:** verteporfin, yes-associated protein/TEA domain inhibitor, non-photoactivated therapy, photodynamic therapy, hippo pathway

## Abstract

Verteporfin (VP) has long been clinically used to treat age-related macular degeneration (AMD) through photodynamic therapy (PDT). Recent studies have reported a significant anti-tumor effect of VP as well. Yes-associated protein (YAP) is a pro-tumorigenic factor that is aberrantly expressed in various cancers and is a central effector of the Hippo signaling pathway that regulates organ size and tumorigenesis. VP can inhibit YAP without photoactivation, along with suppressing autophagy, and downregulating germinal center kinase-like kinase (GLK) and STE20/SPS1-related proline/alanine-rich kinase (SPAK). In addition, VP can induce mitochondrial damage and increase the production of reactive oxygen species (ROS) upon photoactivation, and is an effective photosensitizer (PS) in anti-tumor PDT. We have reviewed the direct and adjuvant therapeutic action of VP as a PS, and its YAP/TEA domain (TEAD)-dependent and independent pharmacological effects in the absence of light activation against cancer cells and solid tumors. Based on the present evidence, VP may be repositioned as a promising anti-cancer chemotherapeutic and adjuvant drug.

## Introduction

Verteporfin causes photochemical damage to the mitochondria *via* ROS accumulation when activated by a 690 nm laser, it is routinely used as a PS for treating AMD (Mellish and Brown, 2001). Although PS was initially tested in anti-cancer PDT, the lack of effective tumor targeting limited its applications since the efficacy of PDT depends on the selective accumulation of photosensitizing drugs in tumor tissues (Mccaughan, 1999), however, the development of laser technology and innovative drug-loaded nanomaterials have re-emphasized the role of VP in tumor photoablation (Baskaran et al., 2018). In addition, VP exerts multiple YAP/TEAD pathway-dependent and independent effects on tissue regeneration, inflammation, and tumor development even in the absence of photoactivation (Gibault et al., 2016; Hertig et al., 2017; Wang Y. et al., 2019). In this review, we have summarized the pharmacological effects of VP on tumors both in the presence and absence of photo-stimulation, in order to provide new insights into anti-cancer chemotherapeutic drug design and targeted therapy.

## The Role of Verteporfin as a Photosensitizer in Tumors

Visudyne is the second-generation PS liposomal VP approved by the FDA of the United States in 2000. VP was passively loaded onto the lipid membrane of liposomes by repeated freeze-thaw method. VP could specifically accumulate into the neovascularization by using liposome and binding with apolipoprotein. Compared with free drugs, it shows higher degree of PS activity (Scott and Goa, 2000). When VP liposome was first used to cure AMD, it showed a strong anti-angiogenesis effect, which means that it could also inhibit tumor growth (Li et al., 2020). This part mainly explores the pharmacological effect of VP as PS on solid tumors under light activation.

**Figure 1:**
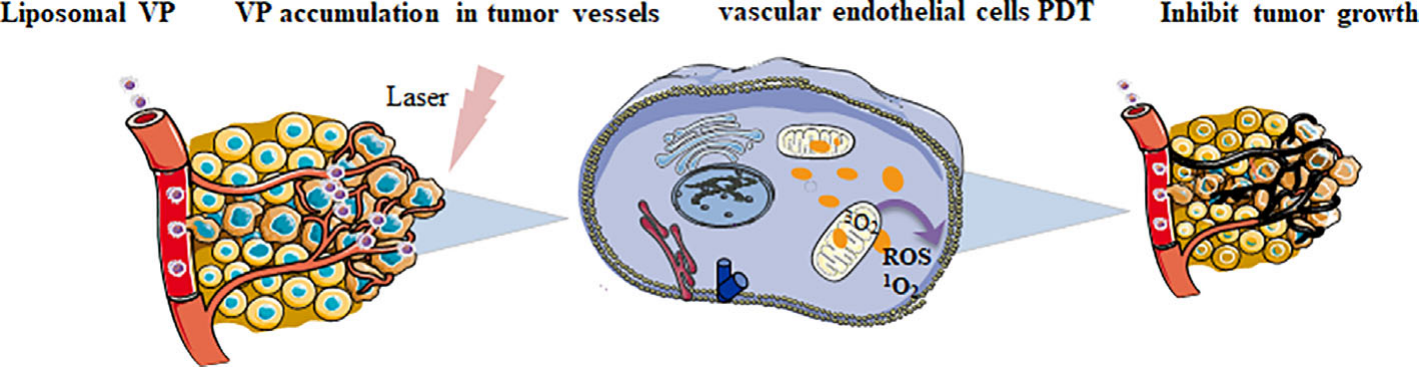
The role of photo-activated liposomal VP on tumor cells.

PDT involves three main elements: cells, blood vessels and immunity (Abrahamse and Hamblin, 2016; Kleinovink et al., 2017). When PS molecules are exposed to light energy, low-energy electrons in singlet state will transit to high-energy electrons in singlet state, and some spontaneously convert to excited triplet state. The electrons in excited triplet state can interact with oxygen, transfer energy to oxygen and produce reactive oxygen species. Cell death is triggered by the complex interaction of autophagy, programmed cell death, apoptosis and necrosis (Kwiatkowski et al., 2018). Vascular targeted photodynamic therapy preferentially targets at abnormal blood vessels by laser irradiation, and delivers PS to the vascular system, causing rapid atrophy and apoptosis of vascular endothelial cells both *in vitro* and *in vivo*. Besides, oxidative damage of vascular endothelial cells can lead to cell loss, and the exposure of vascular basement membrane can result in platelet activation and aggregation, followed by vascular occlusion and blood flow stagnation, the target tissue will be finally destroyed (Ichikawa et al., 2004) (**Figure 1**). The effect of VP PDT on blood vessels depends on several factors: dose, duration, and delivery of light dose and characteristics of tissues (Canal-Fontcuberta et al., 2012). VP PDT selectively damages neovascular endothelial cells, and treats diseases characterized by activation of neovascularization, such as AMD (Scott and Goa, 2000), central serous chorioretinopathy (van Rijssen et al., 2019), polypoid choroidal angiopathy, and choroidal neovascularization (Cohen, 2009). In ocular oncology, PDT has been used for the treatment of localized choroidal hemangioma for more than 10 years, as well as many other ocular tumor diseases, such as choroidal hemangioma, melanoma, choroidal metastasis, retinal capillary hemangioma, and angio-proliferative tumor (Rundle, 2014).

### Effect of Verteporfin as Photosensitizer in Ocular Tumors

PDT with VP as the PS is well recognized for the treatment of retinal/choroidal vascular abnormalities (including intraocular tumors, especially capillary hemangiomas). The treatment is relatively noninvasive, with little collateral damage to adjacent structures (Bakri and Kaiser, 2004). The mechanism of VP PDT in the treatment of chorioretinal diseases is to target at vascular endothelial cells to damage vascular endothelial cells, thus resulting in platelet aggregation, coagulation cascade activation, and microvascular occlusion (Kurohane et al., 2001). Given the high demand for cholesterol in tumor cell division and proliferation, liposomes can enhance the specific targeting and uptake of VP to target cells with high low-density lipoprotein (LDL) receptor expression, such as tumor and neovascular endothelial cells.

Compared with normal choroidal and retinal vessels, VP preferentially accumulates in endothelial cells of abnormal neovascularization (Newman, 2016). Choroidal malignant melanoma is the most common primary intraocular malignant tumor among adults. A single intravenous injection of VP 6 mg/m^2^ and laser PDT (689 nm) with an irradiance of 600 mW/cm2 can be used to treat tumors for 83 s (50 J/cm^2^). Researches show that primary PDT was able to resolve 67% of small amelanotic choroidal melanoma within an average of 5 years, without significant impact on visual acuity. At present, PDT for choroidal melanoma is described as the main treatment method, adjuvant radiotherapy or remedial treatment after radiotherapy failure (Turkoglu et al., 2019). A large number of patients need to be prospectively studied to further understand the effect of PDT on choroidal melanoma. Retinal angio-proliferative tumor as a vascular nodular tumor occurs in the neurosensory retina, accompanied by telangiectasia, lipid exudation, and subretinal fluid. According to the standard treatment of choroidal neovascularization secondary to age-related macular degeneration, VP (6 mg/m^2^) was injected intravenously for 10 min. The standard PDT was applied at 50 J/cm2 at 689 nm within 83 s, which started 5 min after infusion. Although other therapies may be more effective in eradicating tumors, PDT is minimally invasive, easy to obtain and has no obvious side effects for retinal angio-proliferative tumors (Hussain et al., 2015). Choroidal metastasis is the most common intraocular malignant tumor. Due to the poor systemic prognosis of most patients with choroidal metastases, the current treatment methods include external radiotherapy, systemic chemotherapy, hormone therapy, brachytherapy, and enucleation. In some cases, extracorporeal radiotherapy can lead to complications of anterior segment and retina. Chemotherapy and hormone therapy are usually associated with a variety of systemic side effects. PDT with VP is effective in treating choroidal metastases through two mechanisms: the direct tumor is caused by selective cytotoxic activity to malignant cells. It also induces intravascular photo-chromism in vascular endothelial cells supplying the tumor. VP was treated with PDT at a dose of 6 mg/m^2^ and a 689 nm diode laser for 83 s. The results are as follow: among 21 tumors in 13 eyes of 10 patients, 18 tumors (86%) were completely dissolved in subretinal fluid, and 81% of them were flat on ultrasound at follow-up. These data indicate that PDT provides reasonable tumor control and high safety for small choroidal metastases (Chu and El-Annan, 2018). Although PDT has been partially replaced by intravitreal drugs that inhibit vascular endothelial growth, it has lost its wide distribution in the ophthalmology. VP PDT on the other hand is still the standard treatment for choroidal hemangioma and polypoid choroidal angiopathy. PDT is effective for less pigmented choroidal melanoma, retinal vascular proliferation and retinal hemangioma (Ziemssen and Heimann, 2012).

### The Role of Verteporfin as a Photosensitizer in Solid Tumors Other Than the Eyes

Vascular targeted PDT has been used for the treatment of ocular diseases with abnormal activation of blood vessels (Miller, 2019). Preclinical studies found that vascular targeted PDT with VP can effectively induce tumor destruction by causing endothelial cell injury and subsequent vascular dysfunction (He et al., 2008; Khurana et al., 2008; Madar-Balakirski et al., 2010). Therefore, plenty of researches have been carried out on this therapy for the treatment of other types of cancer. *In vivo* fluorescence imaging study of rat prostate tumor model by Chen et al. found that vascular-targeted PDT with VP can induce vascular permeability and thrombosis, and eventually lead to vascular closure and tumor necrosis. Vascular targeted PDT with VP could increase vascular permeability and decrease blood perfusion in a dose and time-dependent manner (Chen et al., 2018). In the study of lung squamous cell carcinoma and osteosarcoma bearing mice, 690 nm laser was used to irradiate KLN205 mouse model and LM8 tumor for 3 h or 15 min before tumor. Doppler ultrasound showed that compared with PDT with 3-h interval, PDT with short drug light interval (a 15-min interval) aimed at tumor vasculature can significantly inhibit tumor angiogenesis. In addition, the tumor tissue was cut off from nutrition and oxygen supply, thus effectively slowing down tumor growth. In the 15-min interval of PDT group, the dead cells showed condensed nuclei around the damaged and ruptured tumor vessels, indicating that the PS was mainly localized in the blood vessels and slightly diffused beyond the boundary (Osaki et al., 2007). Similarly, in the study of parathyroid sarcoma bearing mice and dorsal balloon mice, it was found that PDT with 15-min interval was more effective in blocking the blood flow of tumor neovascularization and inhibiting tumor growth compared with 3-h PDT (Kurohane et al., 2001). These data suggest that the 15-min PDT did a better job in inhibiting tumor growth by destroying endothelial cells in tumor neovascularization rather than through direct cytotoxic effects on tumor cells. Therefore, the combination of cell targeted and vascular targeted PDT may become a new and more effective tumor treatment method.

Light penetration to tissues is very limited, so it is necessary to use interstitial light transmission to treat deep-seated tumors. PDT with VP as the PS is a clinically recognized vascular blocking therapy. Clinical studies found that vascular targeted PDT with local light sources (such as laser fibers) are widely used to eliminate prostate (690 nm, 0.25 mg/kg liposomal VP and received 50 J/cm^2^) (Momma et al., 1998) and pancreatic tumors (0.4 mg/kg VP, 690 nm, 150mW/cm^2^, Huggett et al., 2014). Banerjee et al. reported for the first time the clinical study of PDT in the treatment of primary breast cancer, including intravenous injection of VP (0.4 mg/kg), and then exposure to increasing light dose (20, 30, 40, 50 J) through ultrasound-guided laser fiber delivery. Fifty patients were followed up for 8 months, with the photodynamic effect detected by PDT. The laser fiber inserted by percutaneous positioning needle can directly transmit light energy to the main body of the tumor, which has little impact on the skin and surrounding tissues (Banerjee et al., 2020). In the future, it may become a new possibility for the treatment of breast tumor that has no response to neoadjuvant therapy or slight response.

There are many negatively charged macromolecules such as phosphatidylserine on tumor blood vessels that are lack of glycoprotein coating, thus forming a vascular endothelial cell surface with large negative charge. Cationic liposomes can deliver drugs to tumor neovascular endothelial cells through the long retention effect of electrostatic adsorption and high permeability (Crommelin et al., 2020). VP can be encapsulated into multifunctional drug delivery systems like liposomes (Michy et al., 2019) and nanoparticles (Zhao et al., 2016) in order to increase its solubility, tumor-specific accumulation, cellular uptake, and phototoxicity, and reduce systemic toxicity in the absence of photoactivation. For instance, the liposome-based VP Pluronic^®^ P123/F127 formulation showed high tumor-targeting efficacy, photosensitizing ability and stability, and inhibited MCF-7 and PC3 cancer cell proliferation (100, 300, 500, and 700 J/cm^2^ at 690 nm, VP ranging from 1.0 to 10 × 10^−6^ mol/L) (Pellosi et al., 2017). The VP nanostructured lipid carrier (NLC) selectively accumulated in disseminated ovarian tumor nodules and significantly inhibited tumor growth (2 or 8 mg/kg VP-NLC, 200 J/cm^2^ at 690 nm) (Michy et al., 2019). VP loaded mesoporous silica nanoparticles (VP MSNs) can selectively inhibit the proliferation of highly invasive melanoma cells both *in vitro* and *in vivo* (VP-MSNs 10 μg/mL), the cells were irradiated with a 650/8 filter for different times (0, 30, 60, 120, 180 s) with a plate reader equipped with a standard tungsten-halogen lamp (75 W, spectral range 320–1000 nm). PDT based on VP MSNs does not affect the proliferation of normal human keratinocyte line (HaCaT) or retard the growth of melanoma cell line (A375P) (Rizzi et al., 2017).

### The Role of Verteporfin Chemophotodynamic Therapy in Solid Tumors

Although PDT can induce vascular closure in the internal vessels of tumors and cause extensive death of tumor cells, the survival of tumor cells can be detected around the tumor, which is related to subsequent tumor recurrence (Chen et al., 2018). Multifunctional VP nanoparticles can also be used as an adjuvant to augment the efficacy of chemotherapy drugs and reduce the side effects of chemotherapy drugs. For example, temozolomide used in conjunction with VP nanoparticles markedly decreased the growth of the glioblastoma multiforme U87-MG, T98-G, and U343 cells (VP nano 5.0 μg/ml, TMZ 700, 500, 300 μg/ml, 0.3, 0.7, 1.0 J/cm^2^ at 690 nm) (Pellosi et al., 2019). Kaneko et al. used Hsp90 as a PS target in tumor targeted PDT, and combined Hsp90 small molecule inhibitor with VP to enhance the therapeutic effect of VP in human breast cancer xenografts VP or Hsp90-targeted VP i.p., 25 nmol/mouse, 120 J/cm^2^ at 690 nm). At present, it can be applied to tumors at the depth of several centimeters, such as inflammatory breast cancer (BC) and BC subtype with recurrence of chest wall (Kaneko et al., 2020). Compared to either VP or the anti-angiogenic drug sorafenib (SRB), Pluronic^®^ P123 mixed micelles loaded with VP and SRB significantly inhibited the proliferation of BC MDA-MB-231 cells (SRB: 0-10 μM, VP: 0-0.7μM, 0.75 J/cm^2^ at 690 nm) (Pellosi et al., 2016). Poly (lactic-co-glycolic acid).

(PLGA)-based “smart” nanocarriers carrying VP and low-dose cisplatin also significantly inhibited SKOV3 cell proliferation (NLC-VP, 2or 8 mg/kg, 50 or 200 J/cm^2^ at 690 nm) (Bazylińska et al., 2019). VP, tumor angiogenesis targeted iNGR peptide, and polylactic acid were assembled into iNGR modified VP nanocomposites (iNGR-VP-NA), and docetaxel (DTX) was loaded into hydrophobic core. The obtained iNGR-VP-NA-DTX showed higher cell uptake and stronger cytotoxicity in human umbilical vein endothelial cells and drug-resistant HCT-15 tumor cells *in vitro*, compared with those without laser or VP-NA-DTX without laser. In addition, laser-induced iNGR-VP-NA-DTX enhanced the inhibition of angiogenesis and induced severe apoptosis and necrosis in tumor tissues, but it had little effect on normal areas (DTX ranging from 1 ng/ml to 5 μg/ml; VP ranging from 10 ng/ml to 10 µg/ml, 0.6 J/cm^2^ at 689 nm) (Jiang et al., 2019). VP, VEGF tyrosine kinase inhibitor and cedrinaib were encapsulated in NPS by PDT *via* 690 nm laser irradiation to trigger BPD effect, as well as inhibit cell proliferation by VEGFR interference and growth factor signaling mechanism (Kydd et al., 2018). The combination of three clinically related phosphatidylinositol 3-kinase (PI3K) pathway inhibitors (byl719, bkm120 and bez235) and VP PDT was evaluated. It was found that although all three inhibitors could synergistically enhance the PDT response of endothelial cells, the synergistic effect of PDT and PI3K/mTOR dual inhibitor bez235 was the strongest. Compared with PC-3, SEVC cells were more sensitive to VP PDT and LY294002. PDT combined with bez235 can increase the apoptosis of endothelial cells and induce sustained inhibition of cell proliferation, triggering larger and longer therapeutic response than each single treatment *in vitro* and *in vivo*. Inhibitors of PI3K signal pathway can enhance the therapeutic effect of vascular targeted PDT, especially the oxidative damage of endothelial cells, which selectively destroys vascular function (Kraus et al., 2017). Although both preclinical and clinical studies have demonstrated its effectiveness of tumor eradication and good safety, incomplete vascular closure and angiogenesis are known to the root cause of tumor recurrence after vascular targeted PDT. The combined application of chemotherapy and PS in nano assembly has shown its synergistic anti-tumor effect, which is a potential method for the treatment of drug-resistant cancer. These favorable results indicate that the further development of VP in PDT or the combination of chemotherapeutic drugs is a promising therapy for cancer treatment.

VP vascular targeted PDT preferentially targets abnormal blood vessels by laser irradiation and delivers photosensitizers to the vascular system, causing rapid atrophy and apoptosis of vascular endothelial cells. Oxidative damage to vascular endothelial cells also led to cell loss and vascular basement membrane exposure, resulting in platelet activation and aggregation, followed by vascular occlusion and blood flow stagnation. Finally, the target tissue was destroyed.

## The Role of Non-Photoactivated Verteporfin in Tumors

### The Hippo-YAP-TEAD Signaling

**Figure 2:**
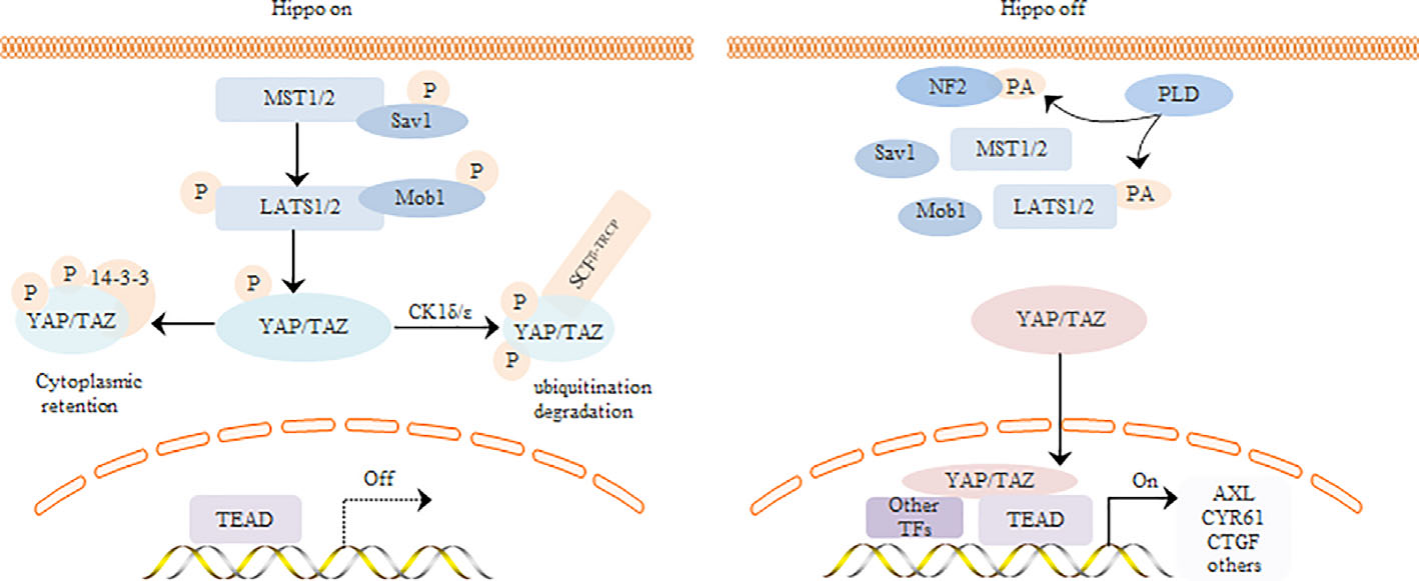
Regulation of YAP/TAZ by core components of Hippo signaling pathway.

The Hippo signaling pathway regulates tissue homeostasis and organ size development in mammals. YAP/TAZ is the core effector of this pathway, and regulates tumor cell proliferation, invasion, and chemoresistance through multiple transcription factors. The core kinase chain of the Hippo signaling pathway phosphorylates YAP and prevents its nuclear translocation. Upon inactivation of the upstream kinase cascade, unphosphorylated YAP is translocated to the nucleus where it functions as a co-transcriptional activator (**Figure 2**). The human YAP gene is located on chromosome 11q22, and the YAP protein contains an N-terminal TEAD-binding domain (TBD), a 14-3-3 protein binding site, and one or two WW domains in the middle. In addition, some YAP isoforms contain the SH3 binding motif, while the C-terminal contains a transcriptional activation domain and a PDZ binding motif. According to the number of WW domains, YAP can be divided into two categories and eight subtypes. TAZ and YAP have similar domain composition except that TAZ lacks the second WW domain, SH3 binding motif, and proline-rich domain (Zhao et al., 2010). Since YAP lacks a DNA binding domain, it needs to bind to transcription factors to activate the downstream genes. The TBD domain of YAP can bind to several transcription factors such as TEADs, Smads, and Klf4, of which the YAP-TEAD interaction is best characterized (Vassilev, 2001).

The oncogenic function of YAP is mainly mediated by its nuclear localization and interaction with TEAD transcription factors. The human TEAD proteins include four subtypes, each with an N-terminal TEA domain, DNA-binding domain, proline-rich region, and C-terminal YAP/TAZ-binding domain. TEAD-YAP binding sites that have been identified include hTEAD1 (209–426)-hYAP (50–171), hTEAD4 (217–434)-hYAP (60–100), hTEAD4 (210–427)-hYAP (47–85) (Gibault et al., 2017). TEADs contain a DNA-binding domain but lack an activation domain, while YAP lacks a DNA-binding domain but contains an activation domain. The YAP-TEAD heterodimeric transcription factor activates proliferation, invasion and adhesion-related genes that promote cancer development and progression (Santucci et al., 2015).

There are two main states in the regulation of YAP/TAZ by the core kinase reaction chain of the Hippo signaling pathway. In the first state, the upstream pathway of Hippo pathway is activated, and the activation complex formed by Mammalian Sterile20-like kinase 1/2 (MST1/2) and its regulatory protein Sav1 can directly phosphorylate lats and Mob1. Moreover, Large tumor suppressor (LATS) and Mob1 activation complexes further phosphorylate the transcription co-activator YAP/TAZ, thus inhibiting YAP/TAZ nuclear entry and initiating downstream target gene expression. In the second state, the upstream Hippo pathway signal is out of order and the kinase cascade is inactivated. Unphosphorylated YAP is therefore transferred to the nucleus and acts as a co-activator of transcription after binding with transcription factors.

### Identification of Verteporfin as a YAP-TEAD Inhibitor and Its Role in Tumors

#### Verteporfin Is a YAP Inhibitor

Liu-Chittenden et al. found VP and protoporphyrin IX (PPIX) could inhibited the transcriptional activity of Gal4-TEAD4 from the Johns Hopkins drug library using the luciferase reporter method and co-IP assay in HEK293 cells. Both drugs blocked the interaction between GAL4-TEAD4 and Ha-YAP proteins and inhibited transcriptional activity of the complex. VP showed a significantly stronger inhibitory effect compared to PPIX at the dosage of 10 mM and colocalized with purified YAP protein *in vitro*. Furthermore, 20 mM VP significantly increased trypsin-mediated cleavage of YAP without affecting TEAD2 lysis, indicating that VP selectively binds to YAP and inhibits the YAP-TEAD complex in the absence of light activation (Liu-Chittenden et al., 2012).

YAP and TEADs are up-regulated in many cancer types, and knocking out either inhibits the proliferation, migration, epithelial mesenchymal transformation (EMT) and oncogenic transformation of cancer cells by blocking transcription of the YAP-TEAD downstream target genes (Gibault et al., 2016; Lin et al., 2017). Given that the YAP-TEAD complex is the final step in the Hippo pathway (Meng et al., 2016), its targeted blocking by VP on the upstream proteins and the potential side effects are smaller. In the following sections, we have summarized the pharmacological effects of non-photoactivated VP on cancer cells (**Table 1**) and animal tumor models (**Table 2**).

**Table 1:** The role of YAP inhibitor verteporfin in solid tumor cells.

Cancer	Cancer cell	VP DoseµM	Cellular Effects	Ref.
Liver Cancer	HepG2	0, 0.75	Decrease expression of TEAD4, YAP, FOXM1, TOP2A, MCM2 KIF20A, Cyclin B1, Cyclin B2, and MAD2	(Weiler et al., 2017)
	HepG2, HuH7	0, 5, 10, 20	Decrease cell growth and mTOR and p-mTOR, ERK, p-ERK, pan RAS protein levels; increase cleaved PARP; lead to proteotoxicity, HMW-p62, oligomerization, autophagic flux interference, and LMP	(Gavini et al., 2019)
	Hepa1-6, HuH7, HepG2, Hep3B	0, 2.5, 5, 10, 20, 40	Limit cell proliferation and colony formation; suppress GLUT1, HK2, ALDOA, and LDHA mRNA levels	(Chen R. et al., 2017)
	BEL/FU, SK-Hep1	0, 50	Decrease cell growth, migration, the protein levels of p-mTOR, p-S6, and p-4E-BP1, and induce cell apoptosis	(Zhou Y. et al., 2019)
Pancreatic ductal adenocarcinoma	PANC-1, SW1990	0, 1, 2, 4, 8	Suppress cell proliferation; arrest cells at the G1 phase and induce apoptosis; upregulate protein expression of c-PARP and Bax; downregulate protein expression of YAP, p-YAP, TEAD, CyclinD1, CyclinE1, Ang2, MMP2, Bcl-2, VE-cadherin, and a-SMA	(Wei et al., 2017)
	AsPC, PANC1	0, 3	Suppress cell proliferation and inhibit cell clonogenic ability	(Zhao et al., 2017)
Gastric cancer	AGS, NCI-N87, MGC-803	0, 1, 2, 5, 10	Suppress cell proliferation in a dose dependent manner and decrease expression of YAP1, CTGF	(Kang et al., 2018)
	MKN45, GC04, MKN74, PDX	0, 0.1, 1, 5	Decreased cell growth, Y/T-TEAD, AREG, CTGF, CYR61, IGFBP3, JAG1, LATS2 transcriptional activity; arrest cells at G0/G1 phase	(Giraud et al., 2020)
Colorectal cancer	HCT8/Tax, HCT15/Tax	0, 10	Decrease cell growth	(Li et al., 2017)
	SW480, HCT116	0, 1, 2, 4	Downregulate expression of PD-L1	(Zhang et al., 2019)
	RKO, HCT116	0, 10	Downregulate protein and mRNA expression of CRY61; inhibit H3K4me1, H3K27ac, CBP enrichment at CYR61 promoter	(Xie et al., 2019)
Endometrial cancer	Ishikawa, AN3CA	0, 1, 5	Decrease cell proliferation and the mRNA expression of p65; downregulate expression of IL-11 and IL-6	(Wang J. et al., 2019)
	HEC-1	0, 0.01	Downregulate expression of CDC23 and BUB1B	(Bang et al., 2019)
	KLE, EFE184, SKUT-2	0, 1.25, 2.5, 5, 10	Inhibit cell proliferation and introduce cell death; decrease YAP, TAZ, GAB2, p-mTOR, p-4EBP1 and p-S6, β-catenin levels	(Wang et al., 2015a)
Breast cancer	MDA-MB-231, MDA-MB-231/taxol	0, 1	Decrease the expression of YAP, TAZ, AXL, CYR61, CTGF; decrease cell proliferation, migration and the expression of YAP, Bcl-2; upregulate expression of E-cadherin, vimentin and BAX	(Li Y. et al., 2018)
	ZNF367-overexpressing MDA-MB-231	0, 10	Increase anoikis-induced cell death and repress the colony formation; decrease the expression of YAP	(Wu et al., 2020)
Lung cancer	PC9, PC9GR	0, 5	Decrease expression of YAP1, p-ERK, Bad, and pS75-Bad	(Wu et al., 2018)
Pleural mesothelioma	211H, H2052, H290	0, 3.5	Reduce YAP protein level, mRNA levels of YAP downstream genes CTGF, AREG	(Zhang et al., 2017)
	Meso-1, NCI-Meso-17	0, 0.5 1, 2, 5	Reduce LATS1, LATS1-P, and YAP1 levels, loss of procaspase 8; increased levels of cleaved caspase 3 and 9, and PARP; suppress MCS cell proliferation, spheroid formation, matrigel invasion, migration, and enhance apoptosis	(Kandasamy et al., 2020)
Glioma	LN229, SNB19	0, 2, 10	Inhibit cell growth; induce phosphorylation of p38 MAPK; reduce c-myc, AXL, Survivin, CYR61, VEGFA, OCT-4, and CTGF levels	(Al-Moujahed et al., 2017)
Myxoid Liposar-coma	MLS 402-91, MLS 1765-92 MLS 2645-94	0, 0.5 1, 1.5 2	Suppress cell proliferation and YAP1, FOXM, PLK1, phosphorylate histone H3^S10^ levels; increase cleaved PARP level; decrease luciferase activity in MLS cell lines co-transfected with a constitutively active YAP1S127A mutant	(Trautmann et al., 2019)
Bladder Cancer	OV6^+^ UMUC3, J82	0, 0.5	Decrease PDGFB expression and PDGF-BB secretion	(Wang J. K. et al., 2019)
Intrahepatic Cholangiocarcinoma	HuCCT1, TKKK	0, 10,20	Decrease expression of YAP, N-cadherin, vimentin, OCT4, STAT3; increase E-cadherin, p-YAP, Akt, p-Akt, mTOR, and p-mTOR level; inhibit CSC-Like property and anoikis resistance	(Sugiura et al., 2019)
Thyroid cancer	NF2-null Cal62, Hth83	0, 0.25, 0.5, 0.75, 1	Inhibit growth of cells, decrease YAP, p-YAP, TEAD, KRAS, HRAS, p-MEK and p-ERK	(Garcia-Rendueles et al., 2015)
Synovial Sarcoma	CME-1, SYO-1	0, 0.25, 0.5, 0.75, 1, 1.25, 1.5, 3	Reduce cell viability; inhibit YAP/TAZ-mediated transcriptional activity and increase apoptosis (cleaved PARP); reduce YAP, TAZ, FOXM1, CTGF and PLK1 protein levels; reduce TEAD luciferase reporter activity	(Chen J. et al., 2017)
Osteosar-coma	U-2OS	0.1-10	Reduce cell viability and migration; inhibit YAP, CYR61, CTGF and CCND1, ROCK2 levels; increase N-cadherin and β-catenin	(Zucchini et al., 2019)
	Saos-2	0, 1, 3, 5	Reduce YAP, FAK397, FAK576 and FAK	(Husari et al., 2019)
Urothelial cancer	BFTC 905	0, 1	Reduce the expression of YAP, COX2, SOX2, NANOG, OCT4	(Ooki et al., 2018)
Uveal Melanoma	92.1, Mel 270, Omm 1, Omm2.3	0, 5, 25	Inhibit cell proliferation, migration, invasion and induce apoptosis; impair the traits of cancer stem-like cells; reduce the expression of YAP, p-YAP, CTGF, CYR61, bcl-2, bcl-X_L_ protein levels and increase c-PARP, c-caspase3, Cyto c, BAX	(Yu et al., 2014)

**Table 2:** The role of YAP inhibitor verteporfin in animal models.

Cancer	Animal model	VP Dose	Animal Effects	Ref.
Liver Cancer	PDX model	100 mg/kg, i.p. every 2 days for 2 weeks	Reduce tumor growth and progression; decrease Ki67, CCNA2, CCNB1, CD31, VEGF-A	(Gavini et al., 2019)
	HuH7 cells injected to nude mice	25 or 50 mg/kg, i.p. every day for 2 weeks	Prevent tumorigenesis; reduce the number/size of HCC nodules, as well as liver weight; decrease serum lactate levels and mRNA expression of GLUT1, HK2, ALDOA, and LDHA	(Chen R. et al., 2017)
	BEL/FU cells injected to Balb/c nude mice	10 mg/kg, i.p., every 3 days for 3 weeks	Reduce tumor growth and the expression of p-mTOR, p-S6	(Zhou Y. et al., 2019)
Pancreatic ductal adenocarcinoma	PDAC xenograft model	100 mg/kg body weight i.p., every 2 days for 3 weeks	Inhibite the tumor growth; downregulate protein expression of YAP, Ki-67, CyclinD, CyclinE, CD31, Ang2, MMP2, a-SMA	(Wei et al., 2017)
	AsPC1 xenograft-bearing nu/nu mice	50 mg/kg i.p., every 2 days for 3 weeks	Decrease tumour volume and weight; reduce protein expression of Ki67, p-ERK, and p-AKT	(Zhao et al., 2017)
Gastric cancer	MKN45 cells xenograft-bearing NSG mice and GC10 PDX model	0.4, 2 mg/kg injected per mouse at the tumor periphery daily for 3 weeks	Decrease expression of CD44, ALDH1, Ki67, PCNA; reduce tumor growth	(Giraud et al., 2020)
Colorectal cancer	HCT15/Tax cells injected to nude mice	10, 20, 20, 30 mg/kg i.p. once every other day for 3 weeks	Downregulate expression of YAP and COX2; reduce tumor growth	(Li et al., 2017)
Endometrial cancer	HEC-1-B GFP cells injected to nude mice	50 mg/kg i.p. 3 times a week for 3 weeks	Downregulate expression of CCRK, CDK2, CyclinD1	(Bang et al., 2019)
Breast cancer	(231R) xenograft model	5 mg/kg i.p. every 2 days for 3 weeks	Reduce tumor growth and YAP1, Ki-67 levels	(Li Y. et al., 2018)
	MDA-MB-231 and MCF-7 cells injected to female BALB/c-nu mice	100 mg/kg body weight i.p. every 2 times a week	Reduce the lung colonization	(Wu et al., 2020)
Lung cancer	miR-630-knockdown PC9 cells injected into nude mice	5 mg/kg i.p. every 3 days	Reduce tumor growth and upregulate the expression of Bad, downregulate YAP1, p-ERK	(Wu et al., 2018)
	A549 and H1299 cells injected to nude mice	subcutaneously injected with 25 mmol/kg twice a week, seven injections in total	Decrease tumor growth	(Zhao et al., 2019)
Mesothelioma cancer	MCS cells (derived from Meso-1 spheroids injected to NSG mic	0, 50 and 100 mg/kg VP 3 times per week	Reduce tumor formation, YAP1, TAZ, TEAD, Slug and Snail levels; reduce procaspase-3, -8, and -9, and PARP; increased cleaved caspase-3 and caspase-9 levels; activate cell apoptosis	(Kandasamy et al., 2020)
Urothelial cancer	UMUC3 or T24-Luc-OV6^+^ cells orthotopic tumor-bearing mice	100 mg/kg ip.	Reduce tumor growth	(Wang J. K. et al., 2019)
Synovial Sarcoma	SYO-1 cells injected to NSG mice or PDX model	75 mg/kg, every other day ip.	Suppress tumor growth, reduce YAP, TAZ, FOXM1, CTGF and PLK1 protein levels	(Chen J. et al., 2017)
Endometrial cancer	SKUT-2 cells injected into nude mice	every 2 days at 45 mg/kg ip. for 2 weeks	Decrease tumor number and size	(Wang et al., 2015a)

#### Effect of Verteporfin on Tumor Cell Proliferation, Drug Resistance, and Tumorigenicity

The Hippo signaling pathway plays an important regulatory role in tumor genesis and development. YAP is abnormally activated in multiple tumors, and associated with increased tumor progression and metastasis, and poor prognosis. Therefore, recent studies have explored YAP as a potential target for tumor treatment (Panciera et al., 2017). Consistent with this, VP inhibits the proliferation of pancreatic ductal carcinoma (Wei et al., 2017), non-small cell lung cancer (Zhao et al., 2019), myxoid liposarcoma (Trautmann et al., 2019), and melanoma (Yu et al., 2014) cells, as well as their xenografts in mice. Helicobacter pylori infection of gastric epithelial cells promotes the nuclear translocation of YAP, which induces EMT and eventually gastric cancer. Studies showed that VP inhibits H. pyroli-induced proliferation, invasion, and metastasis of gastric cancer cells by disrupting the YAP1/TEAD4- Connective Tissue Growth Factor (CTGF) axis and blocking transcription of EMT-related genes (Kang et al., 2018; Li N. et al., 2018). In patients with dedifferentiated liposarcoma (DDLS), the conversion of Tissue Inhibitors Of Metalloproteinase 4 (TIMP-4) to TIMP-1 is associated with poor prognosis.TIMP-1 knockout, TIMP-4 overexpressing, or VP-mediated YAP/TAZ blockade can inhibit the proliferation and migration of DDLS cells (Madhu et al., 2019).

VP also mitigates YAP/TEAD-induced chemoresistance in cancer cells. For instance, VP induced apoptosis in paclitaxel-resistant colon cancer cell lines by downregulating YAP and cox-2, and inhibited growth of the xenografts in mice (Li et al., 2017). In addition, the YAP-TEAD complex promotes transcription of the pro-angiogenic Cysteine-Rich Angiogenic Inducer 61 (CYR61) and the immunosuppressive programmed cell death-Ligand 1 (PD-L1) in colon cancer tissues and cell lines (Xie et al., 2019; Zhang et al., 2019). Gene silencing or VP-mediated inhibition of YAP1 downregulated both factors in colon cancer cells. YAP1 is associated with the early relapse in paclitaxel-resistant patients, and nonlight-activated VP reversed YAP-induced paclitaxel resistance in the HCT-8/T liver cancer cells both *in vitro* and *in vivo* (Pan et al., 2016). Furthermore, VP inhibited the growth of the paclitaxel-resistant breast cancer cell line MDA-MB-231 (Li et al., 2019), and sensitized the HER-2 positive breast cancer cell line HCC1569 to lapatinib (Lin et al., 2015). The clinical stage and multidrug resistance of non-small cell lung cancer (NSCLC) depends on the overexpression of WBP5. VP sensitized the WBP5-overexpressing H69 lung cancer cells to multiple chemotherapy drugs, and decreased their proliferation rate and invasive ability (Tang et al., 2016). VP inhibited tumor cell proliferation *in vitro* and *in vivo* alone or in combination with doxorubicin and pan-RAF inhibitors, and the combination therapy showed greater effect against xenografts (Zhao et al., 2017; Isfort et al., 2019).

YAP1/TAZ-TEAD also plays an important role in maintaining cancer stem cells (CSCs), the major determinants of tumor recurrence, metastasis and chemoresistance, and modulates the expression of CSC markers (Shibata and Hoque, 2019). VP and CA3 weakened spheroid formation, matrix invasion, and tumor formation of the mesothelioma stem cells by inhibiting YAP1/TEAD (Kandasamy et al., 2020). Likewise, Yu et al. found that VP impaired the oncogenic properties of melanoma stem cells (Yu et al., 2014). VP sensitized the OV6+ bladder cancer stem cells to cisplatin by inhibiting the YAP/TEAD1/PDGF-BB/PDGFR autocrine signaling pathway, which downregulated PDGFB and impaired PDGF-BB secretion (Wang J. K. et al., 2019). VP also downregulated the stem cell markers SOX2, NANOG, and OCT4 in transitional cell carcinoma of the bladder when used in combination with COX inhibitor (Ooki et al., 2018). Dissemination of circulating tumor cells is crucial for distant metastasis, and YAP1 increases the number of circulating tumor cells following activation by the chromatin remodeling protein ZNF367. VP downregulated YAP1 in the ZNF367-overexpressing breast cancer MDA-MB-231 and 4T1 cells, and significantly reduced lung metastases in mouse models. It also inhibited the expression of tumor stem-associated protein SOX2, CD44, and CD133 in drug-resistant breast cancer cells and upregulated anoikis-induced cell death in drug-resistant breast cancer and cholangiocarcinoma cell lines (Li et al., 2019; Sugiura et al., 2019; Wu et al., 2020). Taken together, VP can sensitize cancer cells to several chemotherapeutic drugs, including cisplatin, paclitaxel, tyrosine kinase inhibitors (TKIs), and RAF inhibitors by targeting axis. The mechanisms underlying YAP/TEAD-dependent drug resistance need to be elucidated further to treat recalcitrant tumors with greater efficacy.

#### Regulation of Hippo-YAP-Cross Signaling Pathway by Verteporfin

**Figure 3:**
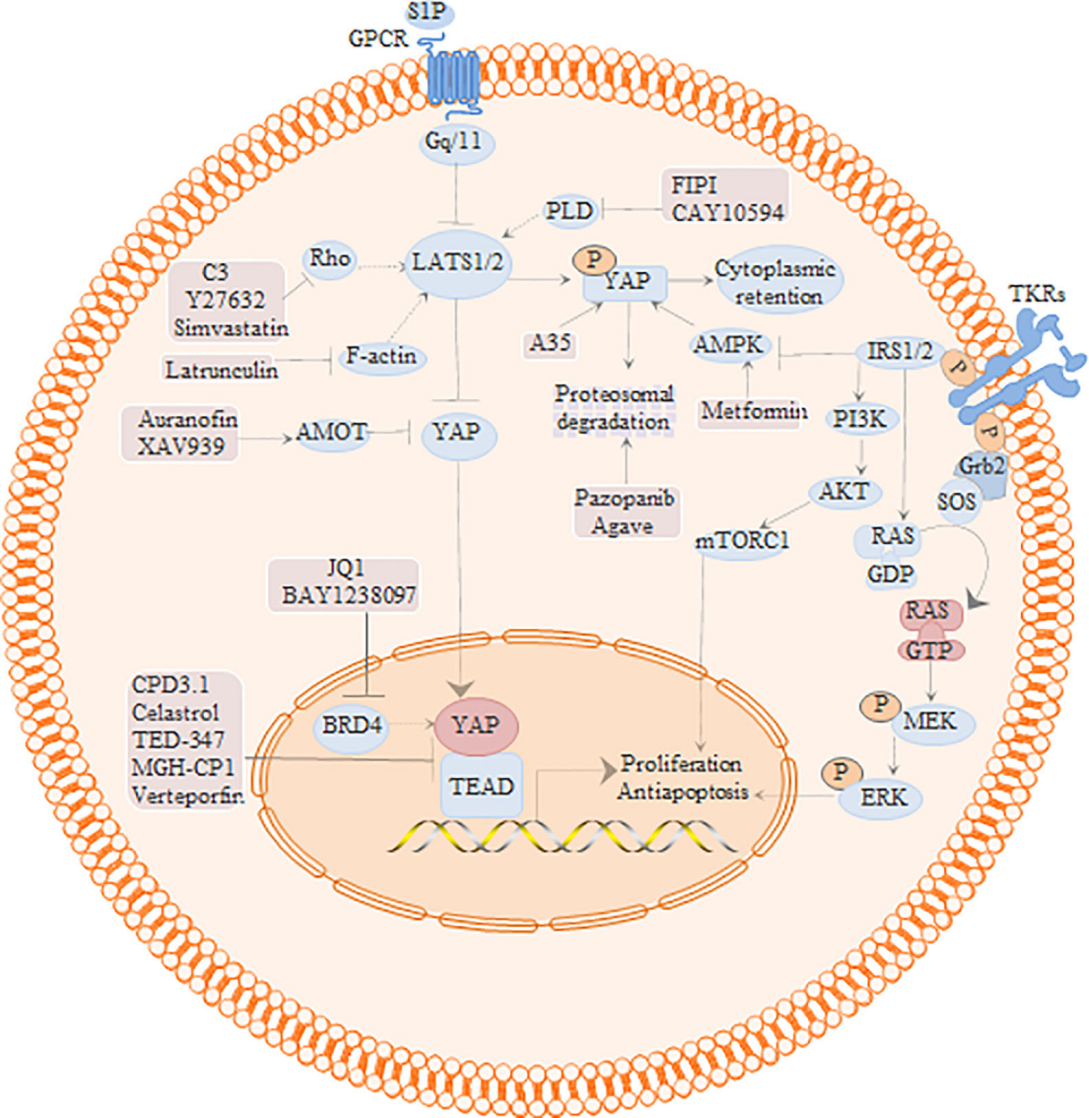
Regulation of Hippo signaling pathway and potential inhibitors of YAP/TEAD.

The YAP-TEAD complex is the central effector of multiple intersecting pathways (**Figure 3**), and is therefore a potential target for cancer treatment. The Hippo/YAP and PI3K pathways interact at multiple levels; for instance, the scaffolding protein GAB2 is a key target of YAP and also interacts with growth factors and the PI3K signaling pathway. VP inhibited GAB2-dependent PI3K/AKT signaling in endometrial cancer cells by inhibiting YAP and TAZ, downregulated p-mTOR and its target genes p-4EBP1 and p-S6 in endometrial and liver cancer cells, and inhibited the growth of the xenografts (Wang et al., 2015a; Zhou Y. et al., 2019). Insulin resistance is an important pathological mechanism of endometrial cancer and endometriosis. PI3K/Akt regulates insulin/IGF1 signaling, and IRS1/2 expression in patients with endometrial cancer is positively correlated with YAP/TAZ. The insulin sensitizer metformin competitively binds to the transcription factor IRF-1 to inhibit the expression of YAP in A549 lung cancer cells. VP augmented the effect of metformin on YAP in lung cancer cells, and reduced the number of tumors in xenograft-bearing mice (Jin et al., 2018). In addition, the transcriptome of VP-treated endometrial cancer cells showed differential expression of 549 genes involved in TGFβ1 regulation, lipoprotein metabolism, cell adhesion, endoderm cell differentiation, and integrin-mediated signaling pathways relative to that in the control cells. YAP inhibitors or metformin alone only partially inhibited the function of insulin and IGF1 in endometrial cancer cells, while their combination completely blocked the effects of insulin (Wang et al., 2016). YAP transcriptionally activates IL-6, and stimulate IL-11 by up-regulating p65. Targeted inhibition of YAP by VP inhibited the binding between YAP and the IL-6 promoter, and downregulated IL-6 and IL-11 in endometrial cancer cells, resulting in lower proliferation rates (Wang J. et al., 2019), increased sensitivity to adriamycin, and 45.36% decrease in tumor weight in the treated mice (Bang et al., 2019).

The RAS-RAF-MEK-ERK signaling pathway regulates tumor cell proliferation, differentiation, invasion, cell cycle, and other processes. The loss of tumor suppressor NF2 in thyroid cancer activates YAP-TEAD transcription through RAS signaling. VP blocked the transcription of KRAS, HRAS and NRAS in the NF2-knockout Cal62 and Hth83 thyroid cancer cells by inhibiting YAP-TEAD, which reduced the proliferation and significantly retarded xenograft growth in mice (Garcia-Rendueles et al., 2015). KRAS/FSTL5 double mutations can desensitize KRAS mutant lung cancer cells to XPO1 inhibitors. Consistent with the fact that FSTL5 mutations are often accompanied by YAP1 activation, the combination of XPO1 inhibitor and VP significantly inhibited the proliferation of these resistant KRAS/FSTL5 double mutant cancer cells (Kim et al., 2016). The mir-630/YAP1/erk feedback loop modulates the resistance of EGFR-mutated tumors to TKIs. Combination therapy with VP and gefitinib inhibited YAP1, p-ERK and ps75-bad in lung cancer cells, and suppressed PC9 mir-630 knockout lung cancer xenografts in nude mice (Wu et al., 2018). Furthermore, VP-mediated inhibition of YAP downregulated FOXM1 and CTGF, and suppressed YAP/TEAD4/FOXM1-dependent activation of CIN-related genes in liver cancer and synovial sarcoma cells (Chen J. et al., 2017; Weiler et al., 2017). In addition, VP also inhibited liver cancer progression by blocking the HMGB1-YAP pathway and inhibiting s1p-mediated YAP upregulation (Zhao et al., 2017; Jung-Chien et al., 2018).

ROCK1 and ROCK2 are the negative regulators of the Hippo tumor suppressor pathway, and while activation of the Rho/Rock signal can increase YAP activity in pleural mesothelioma, ROCK2 knockout decreased tumor volume in a mouse model of osteosarcoma. VP also inhibited the proliferation of osteosarcoma (U-2OS) and mesothelioma (211H, H2052) cell lines by targeting YAP and its target genes, which upregulated N-cadherin and β-catenin (Zhang et al., 2017; Zucchini et al., 2019). Activated YAP is known to stimulate the secretion of FGF ligands, which bind to FGF receptors and activate downstream PI3K, YAP and MAPK pathways. The Hippo/YAP and FGF/FGFR pathways form a positive feedback loop that regulates the activity of advanced ovarian serosa cancer cells. Non-photoactivated VP inhibited YAP, FGF1, and FGF2 in the lats knockout ovarian cancer cells, which induced apoptosis and suppressed their proliferative and migration abilities (Hua et al., 2015). Activation of p38 MAPK induces apoptosis in various tumor cells. Moujahed et al. found that VP inhibited the expression of YAP-TEAD, VEGFA and OCT-4 in the SNB19 and LN229 glioma cells without light activation, and up-regulated p38 MAPK (Al-Moujahed et al., 2017). Taken together, VP inhibits tumor growth and improves chemoresistance by targeting multiple signal transduction pathways associated with YAP-TEAD.

The Hippo/YAP and PI3K pathways interact at multiple levels. PI3K/Akt regulates insulin/IGF1 signal transduction, while RAS-RAF-MEK-ERK signal activates the YAP-teach transcription signal pathway, regulating tumor cell proliferation, differentiation, invasion, cell cycle, and other processes. The activation of Rho/rock signal increases the activity of YAP. Cpd3.1 and VP could directly inhibit YAP-teach interaction. Simvastatin could indirectly inhibit the function of nuclear YAP by acting on the upstream signal pathway of YAP.

### Verteporfin Inhibits Autophagy in Tumors

VP blocked the accumulation of autophagosomes in the breast cancer MCF-7 cells following 1μM (IC50) chloroquine (CQ) treatment or serum starvation (Donohue et al., 2011). Increased autophagy is frequently observed in malignant tumors, and knocking out YAP and TAZ in HeLa cells decreased the number of autophagosomes *via* downregulation of LC3-II and Baf A1. In addition, YAP/TAZ also transcriptionally regulates the expression of F-actin cytoskeleton and myosin II that are critical for autophagosome formation. At high cell densities, Hippo signaling is activated and relocates YAP/TAZ from the nucleus to the cytoplasm, which decreases transcription of myosin II complex and other actin cytoskeleton-related genes, resulting in impaired autophagic protein transport and autophagosome production. Likewise, VP treatment also reduced the expression of myosin light chain 2 in HeLa cells (Pavel et al., 2018).

**Figure 4:**
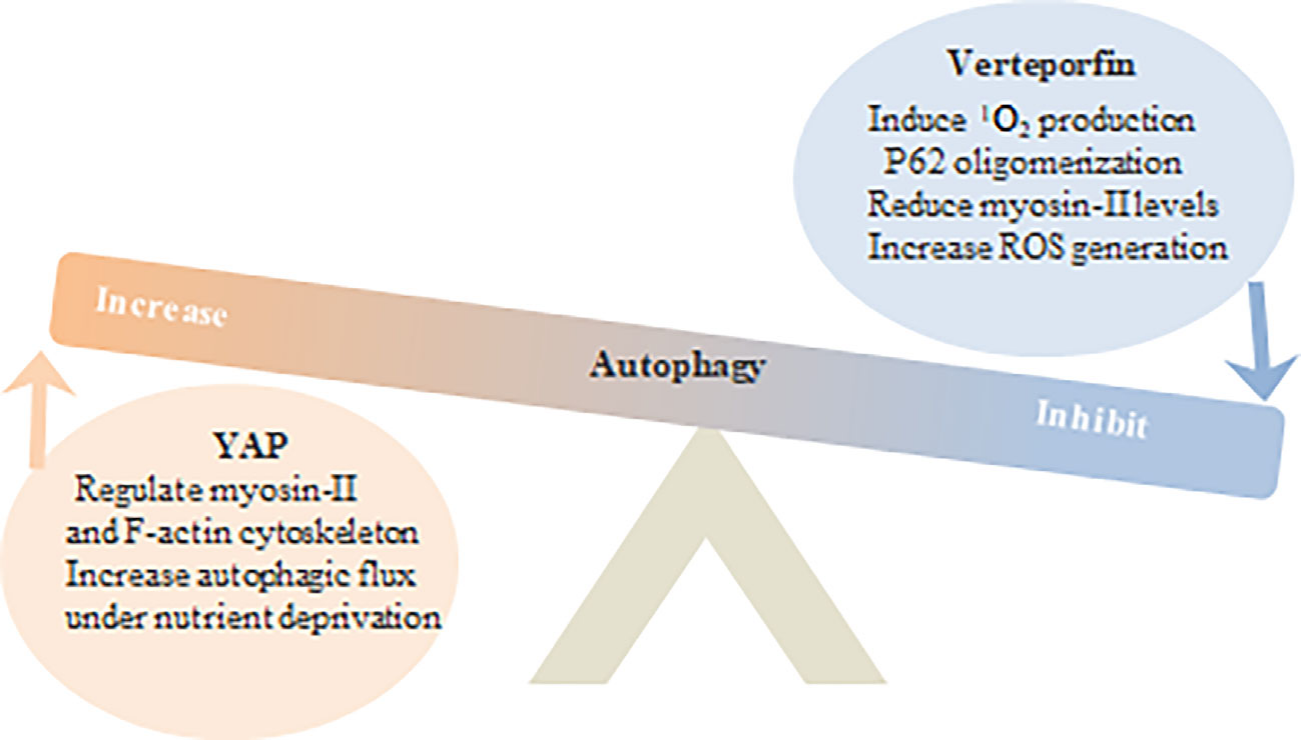
Hippo YAP and VP regulate tumor cell autophagy.

Inhibition of autophagy sensitizes tumor cells to chemotherapeutic drugs, while lysosomal isolation induces chemotherapy resistance. VP alkalizes the pH of lysosomes by reducing RAS expression, which disrupts lysosomal membrane permeabilization and autophagy flux, and increases the expression of HMW-p62 to augment the effect of sorafenib in HepG2 and HuH7 liver cancer cells (Gavini et al., 2019). In the absence of special laser activation, VP inhibits autophagy *via* p62 cross-linkage, which could be a potential target for neutralizing drug resistance in cancer cells (**Figure 4**).

YAP can increase autophagy flux by regulating the cytoskeleton of myosin II and F-actin, or in the condition of nutrient deprivation. VP inhibits autophagy by producing 1O2 and engaging in p62 oligomerization, reducing myosin II levels and increasing reactive oxygen species ROS.

### Cytotoxic Effects of Verteporfin Independent of YAP

The FAK signaling pathway is an upstream regulator of YAP. VP downregulated FAK and p-FAK in hMSCs and human osteosarcoma-derived cells, along with ß1-integrin, paxillin, and zyxin. However, knocking down YAP in hMSCs and Saos cells had the opposite effect on the FAK pathway (Husari et al., 2019). Similarly, YAP knockout in the TKKK cholangiocarcinoma cells did not affect the Akt/mTOR signaling pathway, while VP significantly upregulated p-Akt and p-mTOR in HuCCT1 and TKKK cells. Furthermore, genetic ablation or VP-mediated pharmacological inhibition of YAP inactivated the IL-6/STAT3 signaling and inhibited IL-6-dependent STAT3 phosphorylation. Interestingly, VP also inhibited the IL-6/STAT3 axis in the YAP-low expressing HuH-28 cells (Wu et al., 2020). In addition, VP inhibited HCQ and bafilomycin A1-induced autophagy in PC-3 and LNCaP prostate cancer cells, which increased p62 oligomerization and ROS production, and downregulated Nrf2 (antioxidant) and Bcl-xl (anti-apoptotic) in prostate tumor cells and xenografts, while YAP1 overexpression had no effect on any of these factors (Wang et al., 2018).

Hypoxia is a known inducer of YAP in glioma cells. VP inhibited the proliferation of YAP/TAZ knockout U87 and U343 cells under hypoxic conditions, indicating a YAP-independent function (Eales et al., 2018). The recurrence, metastasis and chemoresistance of gastric cancer is directly related to the cancer stem cells (CSCs). Non-light-activated VP downregulated YAP1/TAZ-TEAD in CD44+ gastric CSCs, which decreased their proliferation and spheroid forming ability *in vitro*, and inhibited growth of both patient-derived and CSC-derived xenografts (Giraud et al., 2020). The low survival rate of gastric cancer is associated with the overexpression of Clusterin protein in gastric CSCs. Although knocking out YAP1 or YAP2 in gastric CSCs had no effect on Clusterin expression, VP significantly inhibited the latter and was more effective against the CSCs compared to the with gastric cancer cells (MGC-803, BGC-823 cells lines) (Xiong et al., 2019). Similarly, VP downregulated YAP target genes in malignant pleural mesothelioma (MPM) cells and inhibited their proliferation independent of YAP1 knockout (Tranchant et al., 2018). Kuramoto et al. found that VP reduced oxidative phosphorylation in glioma stem cells (GSCs) and decreased mitochondrial membrane potential (MMP) and ATP levels, leading to massive GSCs death. Furthermore, the cytotoxic effect of VP was specific to the GSCs, rather than normal human fibroblasts, mouse astrocytes or rat neural stem cells,independent of YAP and ROS (Kuramoto et al., 2019). To summarize, repositioned VP can also regulate the AKT/mTOR, IL-6/STAT3 and FAK signaling pathways, and inhibit CSCs partially independent of YAP.

## Discussion

YAP-TEAD plays a vital role in cancer development and progression. VP inhibits the interaction between these two factors and subsequent transcription of downstream genes in the absence of photoactivation. Therefore, it is a highly suitable candidate for targeted anti-cancer treatment. Multifunctional nanoparticle carriers loaded with VP can selectively kill tumor cells under photoexcitation, whereas non-photoactivated VP inhibits YAP, SPAK, and OSR1 by targeting their kinase domains in an ATP-dependent manner and the suppression is more obvious in the dark (Alamri et al., 2018). Furthermore, non-photoactivated VP also inhibited IL-17A production by the AhR-RORγt complex by targeting GLK, which is suggestive of a potential therapeutic effect against Th17-mediated autoimmune diseases (Chuang and Tan, 2019).

There are several concerns regarding the photodynamic effects of VP on tumor cells. First, it is unclear whether laser activation of VP can alter YAP protein expression in tumor cells since singlet oxygen-induced protein cross-linking can only be observed under intense light. Bae et al. showed that only short-term exposure of VP induced protein cross-linking under incandescent light (Bae et al., 2008). In addition, Donohue1 et al. found that low thermal energy can activate VP to generate singlet oxygen in the dark, which covalently cross-links p62 into oligomers (high-MW p62) through oxidative stress carbonylation. Furthermore, the inhibitory effect of VP on p62 and autophagy can be amplified by exposure to overhead laboratory light during cell lysis (Donohue et al., 2014). However, some studies show that photoactivation of VP is necessary for p62 cross-linkage. In human uveal melanoma cells (MEL 270), human embryonic kidney cells (HEK), and breast cancer cells (MCF-7), VP-induced HMW-p62 requires the presence of light (Konstantinou et al., 2017). Interestingly, VP upregulated the expression of 14-3-3σ protein in EFE184 endometrial cancer cells and isolated YAP in the cytoplasm, and this effect was not reversed by knocking out YAP (Wang et al., 2015b). The 14-3-3 family proteins are involved in regulating autophagy (Wang et al., 2012), and the mechanism through which VP induces autophagy in the absence of light remains to be elucidated.

Secondly, When the PS is exposed to light, PS in normal tissues will also be activated, resulting in phototoxicity. However, the half-life (2~3 h) of VP was low, it is also recommended that animals should be left in the dark for 4 h after VP injection to avoid photosensitivity (Gibault et al., 2016). Donohue et al. kept animals in dark until the morning after VP treatment (Donohue et al., 2013). Curry et al. kept all mice in dark for 24 h after VP injection. A particularly detailed toxicity research on VP *in vivo* experiment remains to be studied (Curry et al., 2017).

**Figure 5:**
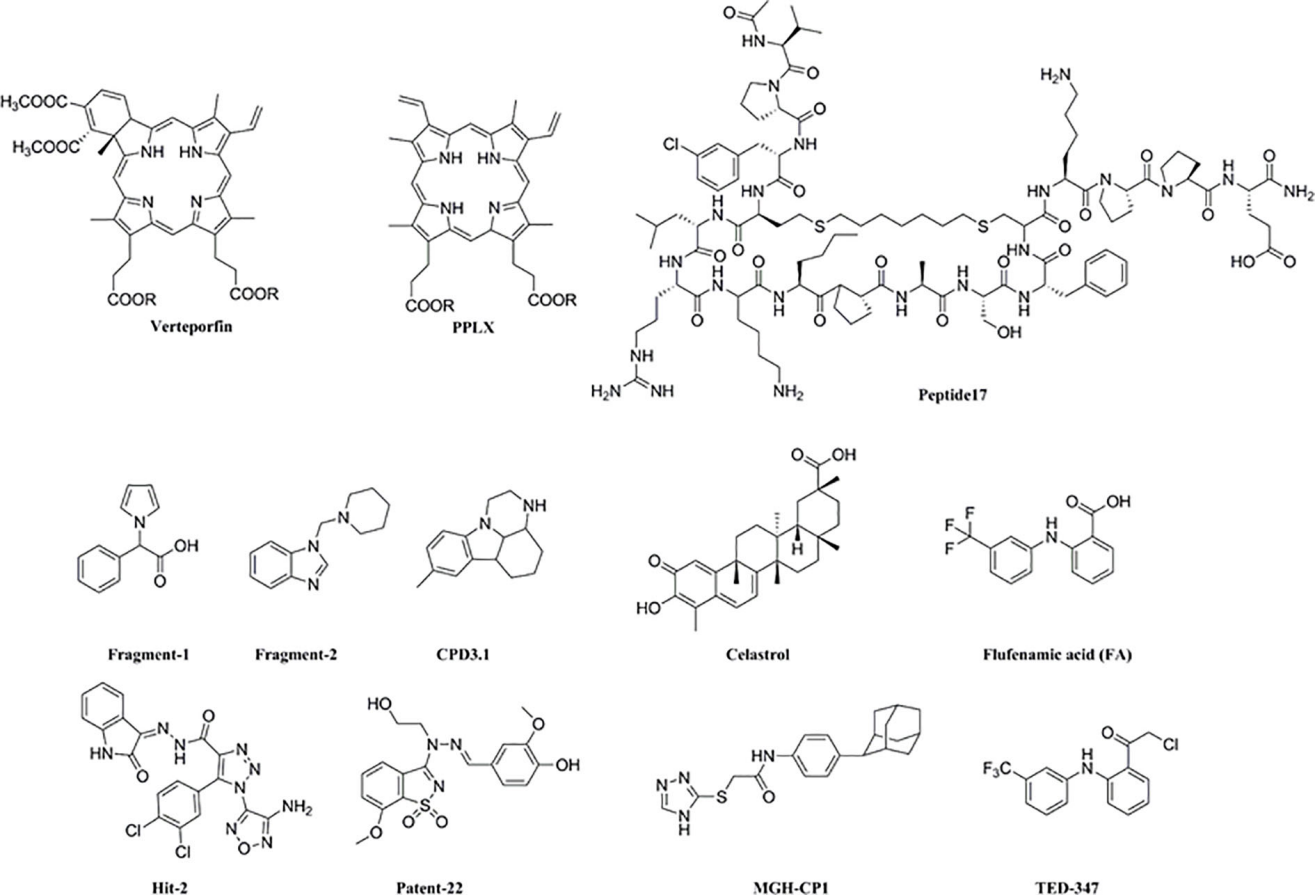
Structural of YAP/TEAD small molecule inhibitors.

Thirdly, YAP/TEAD activity can be impaired by directly blocking the interaction between both proteins (VP and other small molecule inhibitors), or indirectly by inhibiting the upstream factors of YAP like Tankyrase inhibitor (XAV939) or its nuclear localization (dasatinib, pazopani and A35) (Tang et al., 2019). Furthermore, the YAP-TEAD complex has three highly conserved TEAD-YAP binding domain (YBD) interfaces. Interface 1 is an anti-parallel β-sheet formed between the YAP 52-58 amino acids and the β-sandwich of TEAD, interface 2 consists of the LXXLF α-helix motif (YAP residues 61-73) fitted into the hydrophobic groove of TEAD 2, and the third interface comprises of a Ω-shaped side chain formed by YAP residues 86-100 that is inserted into TEAD (Gibault et al., 2017). “Peptide17” (P17) ring YAP-like peptide competes with YAP to occupy the TEAD-YBD interface 3 and disrupts the YAP-TEAD complex. However, peptides are limited by low chemical and physical stability and short half-life in the plasma. Kaan et al. screened the Maybridge Ro3 fragment library through thermal displacement analysis and identified that fragment 1 can down-regulate TEAD luciferin reporter activity in HEK293 cells. X-ray crystallographic analysis revealed that fragment 1 (Kaan et al., 2017) bound to mTEAD4. Fragment 2 (Li Y. et al., 2018) was identified by NMR as the binding partner of the TEAD N-terminal omega loop region, and Patent-22 (Zhou W. et al., 2019) can occupy the TEAD-YBD interface 3 and disrupt the YAP-TEAD complex. Using similar structural analysis methods, Hit-2 (Gibault et al., 2018) and small molecule compounds (CPD3.1) were identified that disrupted the YAP-TEAD interaction. In addition, Hit-2 also inhibited the YAP target gene AXL, Cyr61, and CTGF in MDA-MB-231 cells, and CPD3.1 in HeLa cells, which reduced their proliferation and migration (Smith et al., 2019). Celastrol inhibited the proliferation, migration and clonal expression of the H1299 lung cancer cells and triple-negative breast cancer MDA-MB-231 cells by targeting the YAP-TEAD interactions (Nouri et al., 2019). The binding of the lipid pocket of TEAD to palmitoyl ligand is crucial for its folding, stability, and binding to YAP. Flufenamic acid and MGH-CP1 can bind to this lipid pocket and reduce the expression of YAP target genes (Gibault et al., 2017). TED-347 forms a covalent bond with cysteine in the palmitate-binding pocket of TEAD, leading to allosteric inhibition of YAP-TEAD (Bum-Erdene et al., 2018). This reveals a “pharmacological window” for VP action for maximum tumor growth inhibition by disrupting the YAP-TEAD complex (**Figure 5**).

## Conclusion

To summarize, both photoactivated and non-photoactivated VP can inhibit tumor growth, albeit through different pathways. Apart from HIPPO/YAP signaling, VP also targets the Wnt, PI3K, Ras, mTOR and NF-kB signaling pathways. Furthermore, novel drug carriers have achieved selective tumor accumulation of VP and selective killing of tumor cells *via* its photothermal or photodynamic activation. Thus, repositioned VP is a highly promising photosensitizer or YAP-TEAD inhibitor for tumor therapy, adjuvant therapy, PDT, and tumor imaging.

## Author Contributions

CW wrote this manuscript. XL revised the manuscript. All authors contributed to the article and approved the submitted version.

## Funding

This work was supported by funds from the National Natural Science Foundation of China (No 81473687), Shandong Provincial Natural Science Foundation, China (No ZR2009CM039 and No ZR2013HM038). High level project cultivation program of Shandong First Medical University, China (No 2018GCC14). Academic promotion of Shandong First Medical University, China (grant no. 2019QL017).

## Conflict of Interest

The authors declare that the research was conducted in the absence of any commercial or financial relationships that could be construed as a potential conflict of interest.

## Abbreviations

VP, Verteporfin; PDT, photodynamic therapy; YAP, Yes-associated protein; GLK, germinal center kinase-like kinase; SPAK, STE20/SPS1-related proline/alanine-rich kinase; ROS, reactive oxygen species; AMD, age-related macular degeneration; LDL, low-density lipoprotein; NLC, nanostructured lipid carrier; MSNs, mesoporous silica nanoparticles; BC, breast cancer; SRB, sorafenib; PLGA, Poly (lactic-co-glycolic acid); DTX, docetaxel; PI3K, phosphatidylinositol 3-kinase; TEAD, TEA domain; TBD, TEAD-binding domain; MST1/2, Mammalian Sterile20-like kinase 1/2; LATS, Large tumor suppressor; PPIX, protoporphyrin IX; EMT, epithelial mesenchymal transformation; CTGF, Connective Tissue Growth Factor; TIMP, Tissue Inhibitors Of Metalloproteinase; CYR61, Cysteine-Rich Angiogenic Inducer 61; PD-L1, programmed cell death-Ligand 1; NSCLC, non-small cell lung cancer; CSCs, cancer stem cells; TKIs, tyrosine kinase inhibitors; PS, photosensitizer.
